# Long-term effectiveness, adherence and safety of twice-yearly paliperidone-palmitate long acting-injectable in patients with schizophrenia in Europe: 2-year mirror-image data from the paliperdone-2 per year study (P2Y)

**DOI:** 10.3389/fpsyt.2025.1540213

**Published:** 2025-03-04

**Authors:** Sofía Manchado Perero, Ana Rodríguez Lorente, Alba García-Pérez, Guillermo Isidro García, Luis Alberto Forcen-Muñoz, Santiago Ovejero García, Rocío Sáez Povedano, Ana Luisa González-Galdámez, Laura Mata Iturralde, Mariluz Ramirez Bonilla, Paloma Fuentes-Pérez, Claudia Ovejas-Catalán, Paula Suárez-Pinilla, Blanca Fernández Abascal, Miguel Omaña Colmenares, María Pilar Campos-Navarro, Enrique Baca-García, Ana Lara Fernández, Sergio Benavente-López, Alberto Raya Platero, Miguel Barberán Navalón, Sergio Sánchez-Alonso, Javier Vázquez-Bourgon, Sofia Pappa, Juan Antonio García-Carmona

**Affiliations:** ^1^ Department of Psychiatry, Fundación Jiménez Díaz University Hospital, Madrid, Spain; ^2^ Department of Psychiatry, Santa Lucía University Hospital, Cartagena, Murcia, Spain; ^3^ Centre of Mental Health Molina de Segura, Molina de Segura, Murcia, Spain; ^4^ Department of Psychiatry, Marqués de Valdecilla University Hospital, Universidad de Cantabria, Santander, Spain; ^5^ Psychiatry and Mental Health Research Group, Instituto Investigación Sanitaria Valdecilla (IDIVAL), Santander, Spain; ^6^ Centre of Mental Health Lorca, Lorca, Murcia, Spain; ^7^ Department of Psychiatry, General Hospital de Villarrobledo, Villarrobledo, Albacete, Spain; ^8^ Department of Psychiatry, Los Arcos-Mar Menor University Hospital, San Javier, Murcia, Spain; ^9^ Department of Psychiatry, Alcanyis General Hospital, Xátiva, Valencia, Spain; ^10^ Department Psychiatry, Infanta Elena University Hospital, Madrid, Spain; ^11^ Department of Psychiatry, San Cecilio Clinic Hospital, Granada, Spain; ^12^ Centro de Investigación Biomédica en Red en Salud Mental (CIBERSAM), Instituto de Salud Carlos III, Sevilla, Spain; ^13^ West London National Health System (NHS) Trust, London, United Kingdom; ^14^ Department of Brain Sciences, Imperial College of London, London, United Kingdom; ^15^ Department of Neurology, Santa Lucía University Hospital, Cartagena, Murcia, Spain; ^16^ Group of Clinical and Experimental Pharmacology, Institute for Biomedical Research of Murcia (IMIB), Murcia, Spain; ^17^ Faculty of Pharmacy and Nutrition, San Antonio Catholic University of Murcia (UCAM), Murcia, Spain

**Keywords:** paliperidone-palmitate 6-monthly, schizophrenia, long-acting injectable antipsychotics, mirror-image study, hospital admission, CGI (Clinical Global Impressions), side effect analysis

## Abstract

**Background:**

LAIs with longer dosing intervals appear to be associated with improved clinical outcomes and added real-world benefits in the management of schizophrenia. Paliperidone palmitate six-monthly (PP6M) LAI provides the longest dosing interval, twice-yearly dosing, among all currently available LAIs. In clinical trials PP6M was found to be non-inferior in preventing relapses in patients with schizophrenia compared to the three monthly formulation (PP3M) though real world data remain limited. Therefore, the aim of this study was to evaluate the acceptability, effectiveness, and safety of PP6M in patients with schizophrenia in real world practice.

**Methods:**

Data were derived from a naturalistic cohort of patients enrolled in the international, multicenter, prospective Paliperidone-2-per Year (P2Y) study. In this 2-year mirror-image study we compare the number of hospital admissions 1 year pre- and post-PP6M initiation as well as the CGI scores at baseline and the point of each PP6M administration. Discontinuation rates and reasons were also collected.

**Results:**

A total of 201 patients (107 outpatients and 94 chronic long-stay inpatients) were included. The majority of patients had switched to PP6M from either PP3M (76%) or PP1M (19%) while the 3% switched from aripiprazole 1-monthly and the 2% from risperidone-LAI and zuclopenthixol-LAI. The mean CGI-Severity score significantly reduced from baseline to the second and third PP6M administrations in the global cohort (2.31 ± 0.14 *vs*. 3.23, p=0.001) as well as in both subgroups. Moreover, the number of hospital admissions decreased from 0.2 ± 0.04 1-year period before to 0.07 ± 0.02 1 year after PP6M initiation (p=0.001). Only 6%, (12 patients, 10 out- and 2 inpatients) discontinued treatment at 1 year of follow-up; Kaplan-Meier curves demonstrated significant differences in PP6M treatment discontinuation between out- and inpatients (p=0.012). The main reason for discontinuation was lack of adherence (5 patients) while only 1 patient stopped treatment due to tolerability issues (extrapyramidal side effects).

**Conclusions:**

This is the first mirror-image study in patients with schizophrenia treated with PP6M in real-world settings showing very high treatment persistence, reduced hospital admissions compared to previous LAIs and no major safety concerns. Our findings suggest that six-monthly treatment with a long-acting antipsychotic may confer additional benefits in the management of schizophrenia. Nonetheless, we were unable to determine the precise changes in symptoms. Therefore, future studies are needed to truly establish the role of PP6M.

## Introduction

Schizophrenia is a severe mental disorder that affects approximately 24 millions of patients worldwide (1). It is characterized by a wide range of symptoms, including cognitive, affective and sensorimotor disturbances alongside positive and negative psychotic symptoms (2). Schizophrenia is associated with broad impairments in cognition, significantly impacting functional recovery and quality of life as well as with higher rates of comorbid mental health disorders such as substance misuse (3). Despite extensive research efforts, the underlying biological and genetic causes of schizophrenia remain poorly understood (4). Dysregulation of neurotransmitters appears to play a crucial role in the pathophysiology of schizophrenia. While dopamine dysregulation has long been a central focus in understanding the disorder, recent evidence suggests that abnormalities in other neurotransmitter systems, such as serotonin, glutamate, gamma-aminobutyric acid (GABA), and acetylcholine, also contribute significantly to the development and progression of the disease (5). Nevertheless, past and current antipsychotic treatments are designed to target and modulate the dopaminergic system.

Long-acting injectable (LAI) antipsychotics represent a significant advancement in the treatment of schizophrenia, particularly but not exclusively, for patients who struggle with medication adherence (6). A recent systematic review found that second-generation antipsychotics (SGAs) were generally associated with improvements in quality of life compared to first-generation antipsychotics (FGAs) (7). The review noted that factors such as side effects, adherence, and personal preferences significantly influence the overall benefits of antipsychotic treatment. Furthermore, the use of long-acting injectable antipsychotics was associated with a stable improvement in the levels of quality of life (7). This effect should be due to the several pharmacokinetic advantages introduced by LAI drugs, which allow for a more stable blood concentration of the drug, with lower rates of side-effects and a better long-term compliance with the treatment. (7, 8).

Paliperidone palmitate six-monthly (PP6M) LAI allows for six-monthly dosing, providing the longest dosing interval, among all currently available LAIs. To date, clinical trials demonstrated that PP6M is non inferior compared to PP3M in preventing relapse in patients with schizophrenia adequately treated with PP1M or PP3M in Western and Asian countries (9, 10). A recent study compared PP6M treatment outcomes between the patients included in the above mentioned clinical trial with a real-world external comparator arm (patients treated with PP1M and PP3M) in the US (11) and showed higher PP6M efficacy in reducing and delaying relapses and improved long-term symptom control compared to PP1M/PP3M in usual-care settings (11). To the best of our knowledge, thus far there is only one published study evaluating the role of PP6M in real world practice. Preliminary findings in patients with a wide variety of diagnoses showed a 94% adherence rate for PP6M at 6 months (12). In this regard real world studies, in particular mirror-image studies, offer several advantages, particularly in evaluating treatment efficacy. By comparing the patient’s condition before and after treatment, these studies control for individual differences, making them a powerful tool for assessing the impact of an intervention. This design minimizes confounding variables, as each patient serves as their own control (13). In the present study we aimed to evaluate the long-term real-world effectiveness, adherence and safety of PP6M in patients with a diagnosis of schizophrenia.

## Methods

The study is conducted in accordance with International Conference on Harmonization Good.

Clinical Practice guidelines, the Declaration of Helsinki and approved by the corresponding Ethics Committee (ref. CEI.23-11_PY2_2022). The protocol and consent forms were approved by the respective institutional ethics committee at each site.

**Figure 1:**
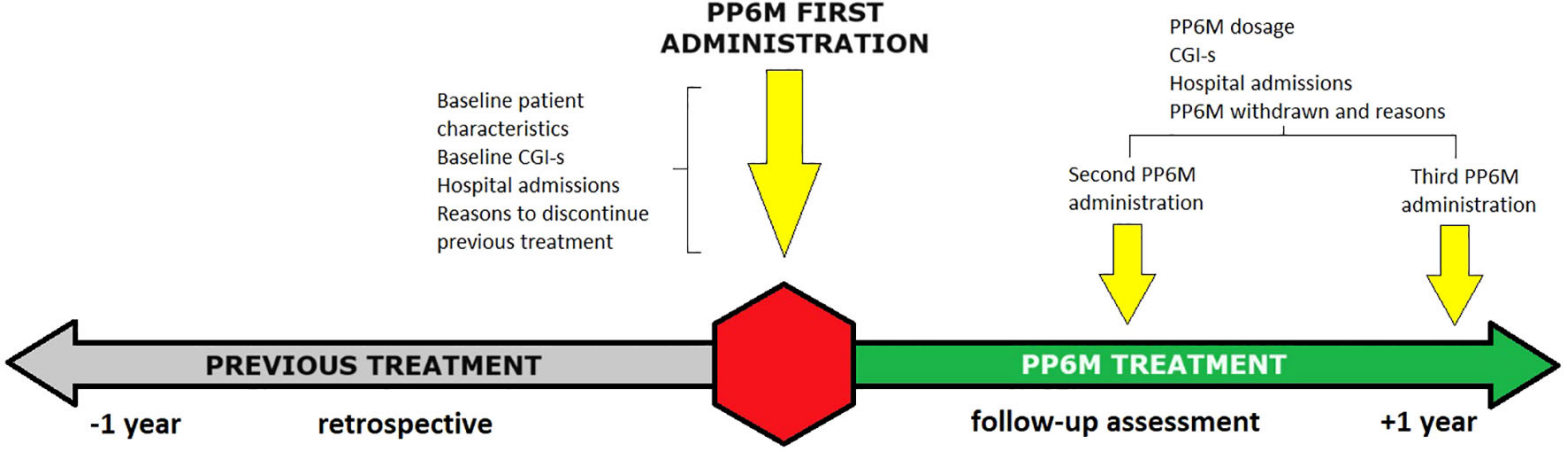
Study timeline scheme.

Data derived from the cohort of patients enrolled in the international, multicenter and prospective mirror-image Paliperidone-2-per Year (P2Y) study. The study protocol was previously published elsewhere (12). Here, we designed a 2-year mirror-image study (**Figure 1**) to determine the effectiveness of PP6M compared to the previous treatment in patients diagnosed with schizophrenia. Adults carrying a diagnosis of schizophrenia, schizoaffective, psychotic, delusional, bipolar or personality disorders, intellectual disability and autism spectrum disorders initiating PP6M treatment were included. Patients <18, pregnant women and those without medical records in the past 2 years were excluded. Data collected extend from 2021 to 2024 and included both outpatients and long-stay inpatients or those in residential care. The index date of the study was defined as the point at which the first dose of PP6M was administered; information was subsequently collected for one year prospectively and one year retrospectively to determine clinical outcomes. Treatment was not predetermined so clinical decisions were taken by the psychiatrists and their patients. There were no exclusion criteria regarding comorbid substance use or affective disorders or other concomitant pharmacological treatments.

### Clinical variables

Demographic and clinical data was collected including sex, age, tobacco and illicit drug use, dosage of PP6M and time interval between doses (in weeks). We also assessed the CGI score at the point of each PP6M administration, collected and compared the number of psychiatric hospital admissions 1 year pre- and post- index date. The CGI is a clinician-assessed scale providing easily understandable measures of patient illness severity for routine clinical practice. It consists of two single-item subscales with intuitive anchor points both rated on a 1-7: one that measures the global severity of symptoms at the time of assessment (CGI-S), and another that assesses global improvement since baseline or the most recent evaluation (CGI-I) (14). We considered treatment discontinuation if the dose of PP6M was not administered within 3 weeks after the expected date for the next dose (up to week 27, included). The reasons for treatment discontinuation were also collected as follows: no adherence, tolerability, ineffectiveness, all-cause mortality or patient, clinician or family preference of other treatment. We recorded the specific side effects if they led to treatment discontinuation, including weight gain, extrapyramidal effects, insomnia, raised prolactin, QTc prolongation, high blood pressure, sedation, digestive effects, hematological disorders, neuroleptic malignant syndrome or other side effects

### Statistical analysis and confounding factors

All analyses were performed using IBM SPSS Statistics version 21.0 (IBM Corp., Armonk, NY, USA). We expressed quantitative variables as means [standard error media] and categorical variables as numbers (percentage). We assessed normality of distributions using histograms and the Shapiro–Wilk test. Patients who withdrew PP6M at 12 months were included in the analysis. Sample basal characteristics were analyzed by Student’s t-test, chi-square and univariate analysis. Variables associated in the univariate analysis and variables with statistical trend (p < 0.1) were entered as factors in a multivariate logistic regression model to identify the risk factors or patients characteristics associated with being initiated with PP6M treatment. Differences with a p value <0.05 were considered significant.

## Results

Here, we present data on patients characteristics, effectiveness and adherence to PP6M compared with the previous treatment up to 1 year after the switch in a naturalistic cohort of patients diagnosed with schizophrenia. A total of 201 patients (107 outpatients and 94 inpatients living in a chronic mental health hospital or residential care home) were recruited as part of the P2Y study and included in this analysis. Sociodemographic and clinical characteristics are outlined in **Table 1** and include the total cohort as well as the two sub-groups separately so as to avoid biases and capture potential differences. To this end, it is worth noting that the outpatients subgroup were significantly younger (48.1 ± 1.3 *vs*. 55.3 ± 1.0, p=0.001), composed by a wide variety of races (p=0.001) in contrast to the long-stay chronic patients that were older and mostly Caucasian (97%). Furthermore, 17% of outpatients were employed compared to only 1% in the chronic inpatient subgroup (p=0.001). 55% of patients in the total cohort smoked tobacco and used other substances without differences between subgroups. Nonetheless, a significantly higher prevalence of tobacco (p=0.016) or cannabis (p=0.041) smokers were found in the inpatients and the outpatients subgroups, respectively. 15% of patients carried a recorded comorbid diagnosis of SUD again without any differences between subgroups. 19% of patients were previously treated with PP1M, 76% switched from PP3M, 3% from aripiprazole 1-monthly (A1M), 2% from other LAIs [risperidone-LAI (RLAI) or zuclopenthixol-LAI (ZLAI)] and 3% from oral antipsychotics. No differences were found in the previous treatment of the patients between subgroups though a statistical trend was noted in the proportion of patients treated previously with PP3M (86% inpatients *vs*. 66% outpatients, p=0.071). The dosage of previous treatments and the reasons for switching to PP6M are summarized in **Table 1**. Higher doses of PP3M were used in the inpatients (620 ± 32mg) compared to the outpatients subgroup (534 ± 28mg, p=0.038). No other dosage differences were found. Among the reasons for switching to PP6M, 39% of patients and 51% of clinicians preferred treatment with PP6M and only 2% reported side effects, 3% no adherence and 4% ineffectiveness of the previous treatment.

**Table 1:** Demographic and clinical data.

	Cohort N=201	Out-patients N=107	In-patients N=94	*p*-value
Sex (%)				0.936
Women	54 (27)	29 (27)	25 (27)	
Men	147 (73)	78 (73)	69 (73)	
Age (y±SEM)	51.4±0.9	48.1±1.3	55.3±1.0	0.001
Race				0.001
Caucassian	180 (90)	89 (83)	91 (97)	
Black	8 (4)	6 (6)	2 (2)	
Latin	9 (4)	8 (7)	1 (1)	
Asiatic	1 (1)	1 (1)	–	
Arabic	3 (1)	3 (3)	–	
Employed (%)	19 (9)	18 (17)	1 (1)	0.001
Tobacco & Drugs (%)	111 (55)	55 (51)	56 (60)	0.429
Tobacco	91 (45)	40 (37)	51 (54)	0.016
Alcohol	34 (17)	23 (21)	11 (12)	0.070
Cocaine	18 (9)	12 (11)	6 (6)	0.243
Cannabis	33 (17)	23 (21)	10 (11)	0.041
Heroine/opiates	3 (1)	2 (2)	1 (1)	0.647
Amphetamines	3 (1)	2 (2)	1 (1)	0.647
SUD	30 (15)	19 (18)	11 (12)	0.231
Previous AP treatment				0.897
PP1M	38 (19)	28 (26)	10 (11)	0.233
PP3M	152 (76)	71 (66)	81 (86)	0.071
A1M	4 (3)	3 (3)	1 (1)	0.970
Other LAIs	3 (2)	2 (2)	1 (1)	0.998
Oral AP	4 (3)	3 (3)	1 (1)	0.970
Dose previous AP treatment (mg±SEM)				
PP1M	175±10	165±12	202±21	0.214
PP3M	579±22	534±28	620±32	0.038
A1M	400±0	400±0	400±0	0.999
Other LAIs	150±50	125±25	200±0	0.873
*R-LAI*	125±25	125±25	–	–
*Z-LAI*	200±0	–	200±0	–
Oral AP				–
*Paliperidone*	9±0	–	9±0	–
*Aripiprazole*	15±0	15±0	–	–
Duration previous treatment (y±SEM)	2.5±0.3	2.3±0.6	2.9±0.2	0.186
Reasons discontinuation previous treatment				
Side effects	4 (2)	3 (3)	1 (1)	0.625
No adherence	6 (3)	6 (6)	–	–
Ineffective	8 (4)	7 (6)	1 (1)	0.405
Patient prefer other LAI	79 (39)	51 (48)	28 (30)	0.042
Family prefer other LAI	1 (1)	1 (1)	–	–
Psychiatrist prefer other treatment	103 (51)	39 (36)	64 (68)	0.001

y=years; SUD, substance use disorder; AP, antipsychotic; LAI, long acting-injectable.

### PP6M initiation

The majority of the outpatient subgroup were initiated with PP6M in a mental health center while only 2 (2%) patients were initiated during a hospital admission. As shown in **Table 2**, the first dose of PP6M was 1068.7 ± 32.1mg. A statistical trend was found between subgroups for inpatients to have been initiated with higher doses compared to outpatients (1125.5 ± 50.9 *vs*. 1018.7 ± 40.1, p=0.097).

**Table 2:** PP6M treatment dosage.

	Cohort N=201	Out-patients N=107	In-patients N=94	
First PP6M administration				
Dose (mg±SEM)	1068.7±32.1	1018.7±40.1	1125.5±50.9	0.097
Second PP6M administration				
Dose (mg±SEM)	962.7±33.8	924.0±38.5	1004.3±56.5	0.236
Interval (weeks±SEM)	23.4±0.2	22.9±0.2	23.9±0.2	0.001
Retention rate (%)	96.0%	93.4%	98.9%	0.286
Third PP6M administration				
Dose (mg±SEM)	995.6±28.3	968.8±37.6	1030.5±42.7	0.281
Interval (weeks±SEM)	23.1±0.2	22.6±0.2	23.7±0.1	0.001
Retention rate (%)	94.0%	90.4%	97.8%	0.135

The second dose of PP6M (962.7 ± 33.8mg) was administered to 96% (N=193) of patients after a mean interval of 23.4 weeks. Moreover, 94% (N=189) of patients were treated with the third dose of PP6M (995.6 ± 28.3mg) after an interval of 23.1 weeks. No statistical differences were found in the second (924 ± 38.5mg in outpatients *vs*. 1004.3 ± 56.5mg in inpatients) and third (968.8 ± 37.6mg in outpatients *vs*. 1030.5.3 ± 42.7mg in inpatients) PP6M mean doses and the retention rate between subgroups for the second (93.4% in outpatients *vs*. 98.9% in inpatients) and third (90.4% in outpatients *vs*. 97.8% in inpatients) administrations. Nonetheless, both the second and third PP6M administrations were given significantly earlier by one week in the outpatients (p=0.001, 22.9 *vs*. 23.9 weeks and p=0.001, 22.6 vs. 23.7 weeks, respectively).

### CGI scores and number of hospital admissions up to 1 year

**Figure 2:**
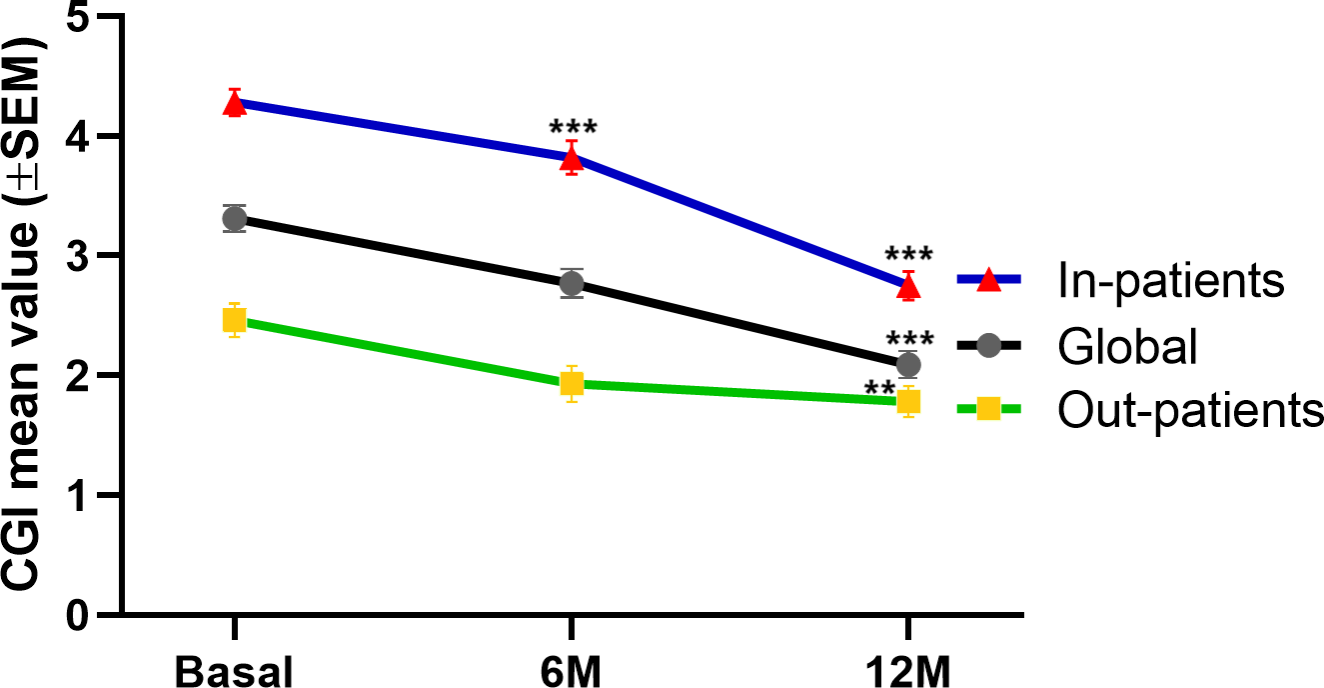
Clinical Global Impressions-Severity (CGI-S) up to 12 months.among in-patients (blue), out-patients (green) and the global cohort (black). Data are expressed as the mean±SEM. Repeated ANOVA test comparing CGI-score 6 and 12 months after PP6M initiation PP6M. ***p<0.001 and **p<0.01 vs. index date.

We assessed the CGI-Severity score at the first (index date), second and third PP6M administration. The mean score decreased from the baseline to the second and third PP6M administrations (**Figure 2**) in the global cohort and in both subgroups. ANOVA with repeated measures showed a significant reduction in the CGI-score at the third administration compared with the index date for the total cohort (2.31 ± 0.14 *vs*. 3.23 ± 0.13, p=0.001). Specifically, in the outpatients group CGI-s score significantly (p=0.01) decreased from 2.36 ± 0.15 at the index date to 1.78 ± 0.14 after 1 year. CGI-s score significantly decreased from baseline (4.02 ± 0.14) to both 6 and 12 months for the inpatients (3.48 ± 0.16, p=0.001; 2.51 ± 0.11, p=0.001, respectively).

**Figure 3:**
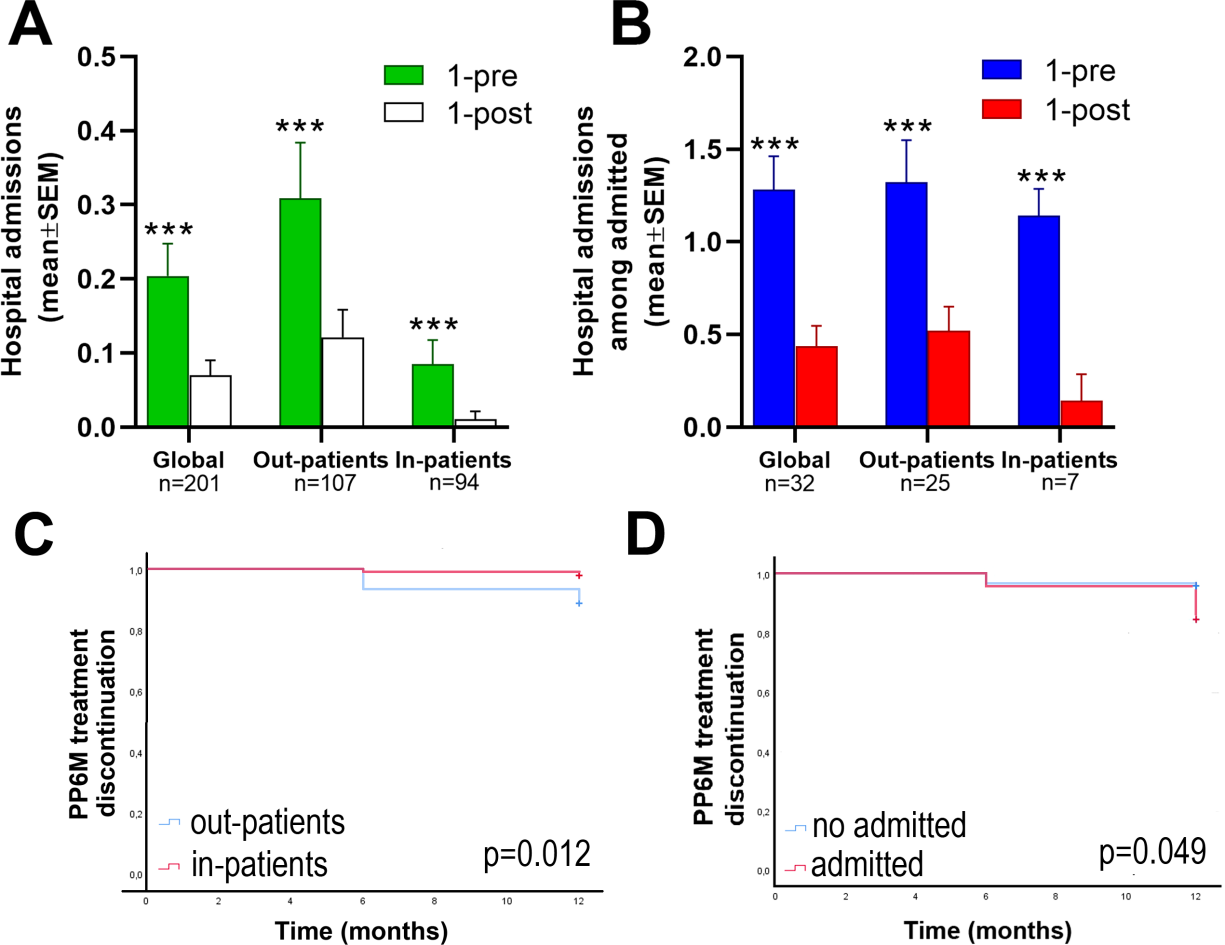
Hospital admissions and treatment discontinuation. Upper is shown the number of hospital admissions. Data are expressed as the mean ± SEM. **(A)** ANOVA test comparing hospital admissions 1 year before and after PP6M initiation in the global cohort and in the out and inpatients groups and in **(B)** including only the patients who were admitted during the 1 year period before initiating PP6M. ***p<0.001 *vs*. 1 year before the index date. Lower is shown PP6M discontinuation: **(C)** Kaplan—Meier curves comparing PP6M treatment discontinuation between out- and in-patients groups. **(D)** Kaplan—Meier curves comparing PP6M treatment discontinuation between out- and in-patients. The p values were obtained from the log rank tests. PP6M =paliperidone 6-month..

Moreover, we collected the number of acute hospital admissions up to 1 year before and 1 year after the index date i.e. the mirror point of PP6M initiation. The number of acute hospital admissions (**Figure 3A**) was expressed as the mean number of hospitalizations per patient per year in the global cohort and both out- and long-stay residential patients subgroups. Only 6 patients (2.9%) were admitted during the 1 year of follow-up. The mean number of hospital admissions significantly (p=0.001) decreased from 0.2 ± 0.04 during the 1-year period before the index date to 0.07 ± 0.02 1 year after treatment with PP6M. This effect was shown in both out- and long-stay groups. Furthermore, 32 patients (16%) were admitted during the year previous to the index date (**Figure 3B**). Similarly, the mean number of hospital admissions significantly (p=0.001) decreased from 1.28 ± 0.18 during the 1-year period before the index date to 0.44 ± 0.11 1 year after treatment with PP6M. This effect was shown in both subgroups.

### Time to and reasons for PP6M discontinuation

We examined PP6M discontinuation at 6 and 12 months after its initiation. A total of 12 (6%) patients discontinued the treatment after 1 year of follow-up. 10 (7%) and 2 (2%) patients in the outpatients and inpatients subgroups, respectively. Furthermore, Kaplan-Meier curves (**Figures 3C, D**) demonstrated significant differences in PP6M treatment discontinuation after 1 year between out- and inpatients (7% CI95%=6.7-7.4 vs. 2% CI95%=1.8-2.2, p=0.012) and between those patients admitted or not before the index date (16% CI95%=10.9-19.7 vs. 4% CI95%=2.6-5.8, p=0.049).

In **Table 3** are summarized the reasons for PP6M discontinuation up to 1 year. The main reason was no adherence to the treatment in the outpatients subgroup (5 patients, 4.6% of the subgroup). Other reasons were as follows: 3 (1.5%) patients preferred other treatment, 2 (1%) patients died by causes not related to PP6M or to mental health disorders, 1 (0.5%) psychiatrist preferred other treatment and only 1 (0.5%) patient stopped because of extrapyramidal side effects.

**Table 3:** All-cause PP6M treatment discontinuation up to 12 months.

	Cohort N=201	Out-patients N=107	In-patients N=94
Total PP6M discontinuation at 1 year	12 (6)	10 (9)	2 (2)
Side effects			
Weight-gain	-	-	-
Extrapyramidal	1 (0.5)	1 (1)	-
Prolactin increase	-	-	-
QTc prolongation	-	-	-
High blood pressure	-	-	-
Sedation	-	-	-
Insomnia	-	-	-
Digestive effects	-	-	-
Haemathological effects	-	-	-
Neuroleptical malignant syndrome	-	-	-
No adherence	5 (2.5)	5 (4.6)	-
Ineffective	-	-	-
Patient died*	2 (1)	1 (1)	1 (1)
Patient prefer other treatment	3 (1.5)	2 (2)	1 (1)
Family prefer other treatment	-	-	-
Psychiatrist prefer other treatment	1 (0.5)	1 (1)	-

*Not related to the treatment or the mental disorder.

## Discussion

The P2Y observational, 4-year mirror-image study is being conducted at multiple sites in Europe in order to capture the wider clinical use and application of PP6M. Here, we present real world data about the use of PP6M in clinical practice including treatment discontinuation and its reasons for the first 201 patients diagnosed with schizophrenia and initiated on PP6M across multiple European settings. To our knowledge, this is the first mirror image study of PP6M with a 1-year follow-up period. In contrast with previous clinical trials (9) carried out with PP6M in patients diagnosed with schizophrenia here we included patients with a variety of comorbidities including substance misuse, and physical comorbidities, such as metabolic syndrome and cardiovascular disease as well as patients with concomitant use of mood stabilizers or antidepressants and other antipsychotics to better reflect pragmatic clinical conditions.

The overall compliance of 94% with PP6M was remarkable in this naturalistic cohort 1 year after treatment initiation. In fact, 90.4% of outpatients and 97.8% of inpatients in our cohort received the third PP6M administration. In the *post hoc* analysis of two clinical trials in European and Asian cohorts, authors compared discontinuation between a group of patients treated with PP6M and PP3M during 1 year and found a similar retention rate (89% and 87%) for PP6M after 12 months treatment in outpatient settings (10, 15). Therefore, our results are overall in agreement with data from clinical trials demonstrating high rates of PP6M compliance in European settings after 1 year or slightly more favorable particularly for the chronic inpatient group. Furthermore, the treatment persistence appears to be much more favorable compared to PP1M; for example an independent study evaluating the long-term real world effectiveness and acceptance of PP1M, reported that 79.5% of patients with schizophrenia continued treatment at one year post-PP1M initiation (16).

Given that most of the patients in our cohort were previously treated with PP3M (and less so with PP1M) for at least 1 year, our findings suggest that adherence in schizophrenia improves when treatment allows for a longer duration of plasma levels and therefore less frequent treatment administrations (17–19). Moreover, there is a growing body of evidence suggesting that even after discontinuing medication, there is a lower risk of relapse in long-acting antipsychotic treatment compared to oral treatment (20, 21). In this regard, further studies are needed to establish the role of PP6M compared to oral and other LAIs antipsychotics.

Furthermore, only 1 (0.5%) patient reported adverse effects (i.e. extrapyramidal side effects) as the reason to discontinue PP6M treatment. Similarly, in a previous clinical trial only 1% of patients reported side effects as the reason to withdraw from treatment (15). 5 outpatients (4.6%) were non-adherent to PP6M and missed more than 4 weeks of the scheduled administration.

Previous data derived from open-label extension clinical trials showed that 121 patients treated with PP-6M remained clinically stable as evidenced by PANSS and CGI-S scores over the 2-year follow-up period (22). In contrast, our results showed a significant decrease in CGI-s score after 1 year of PP6M treatment. Strikingly, this reduction in the CGI-s was more pronounced in the long-stay subgroup. This difference may be explained by several factors such as 1) greater subjective improvement component since the patients in our cohort are more heterogeneous than in clinical trials, 2) shorter follow-up period in our study and 3) better compliance in our sample. Nonetheless, further studies specifically designed are needed to evaluate the potential clinical improvement with PP6M compared to other LAIs.

The aforementioned clinical trials also evaluated the number of relapses in patients diagnosed with schizophrenia and treated with PP6M though did not separately record on the number of psychiatric hospital admissions. Relapse in these studies was defined and measured as time to first occurrence of at least 1 of the following events: psychiatric hospital admission, emergency room visits for worsening symptoms, suicide attempt or injury to another person or significant property damage. Nonetheless, a recent study derived from a clinical trial reported that 2.2% of patients were admitted during the 2-year follow-up period (23). In this regard, 2.9% of the patients in our cohort were admitted during 1 year of follow-up and given the real world conditions of our study, a slightly higher percentage of admissions should be expected in our cohort after 2 years. In clinical trials PP6M demonstrated to be non-inferior compared to PP3M and ensured the maintenance of clinical efficacy in the long term. It is noteworthy however, that our results demonstrated that PP6M reduced hospital admissions compared to the previous antipsychotic treatment, mainly PP3M, in those patients who were previously admitted.

Last, it could be encouraging to confirm in future studies with PP6M some of the clinical effects demonstrated with its predecessors. In this regard, PP1M significantly improved non-core symptoms, which include negative symptoms and cognitive impairments, thereby suggesting that PP6M could have a broader therapeutic benefit that extends beyond symptom management to impact overall quality of life (QoL) (24). This ties into the findings of Sampogna et al. (7), who highlighted the positive effects of second-generation antipsychotic treatment, in particular LAIs, on QoL in patients with schizophrenia. Further strengthening the case for PP6M, Besana et al. (25) highlighted that LAIs may play a significant role in reducing the risk of readmission in first-episode psychosis patients. The study found that those receiving LAI treatment had lower rates of rehospitalization compared to patients on oral medications, emphasizing the potential for these medications to influence the long-term trajectory of early psychosis. In practical terms, reduced readmissions, fewer relapses, and better adherence with PP6M would likely lead to lower hospitalization rates, which are a significant economic burden in schizophrenia management (1). Furthermore, by improving QoL and functional outcomes, PP6M may reduce the need for extensive social support and long-term care, which could help optimize healthcare resource utilization. Therefore, future studies are needed to evaluate this possible additional benefit of PP6M compared to other LAIs in preventing hospital admissions, relapses and its impact on the quality of life of patients.

## Limitations

Several limitations of our study need to be considered. First, although the number of subjects included in this study (n = 201) was relatively large, the size may still be insufficient to fully represent the entire population of patients with schizophrenia. Second, since we only included data collected from clinical practice, we were unable to assess specific symptom scale scores, making it challenging to determine the precise changes in symptoms. Third, while using a mirror-image study design may ensure that some patient specific variables can be controlled and remain constant, it cannot account for other important factors such as changes in socioeconomic variables, patients age or differences in prescribing practices among different regions or individual practice among clinicians, including the shared and supported decision making process. Forth, selection bias was minimized by including a consecutive sample of eligible patients prescribed PP6M at the discretion of clinicians in the absence of any restrictive or prescriptive practice.

## Conclusions

This is the first mirror-image study in patients with schizophrenia treated with PP6M in both real-world settings, out- and in-patients, showing high treatment persistence and no major safety concerns, as well as reduced hospital admissions compared with previous antipsychotic treatments, mainly PP-3M LAI. Our findings suggest that six-monthly treatment with PP may have additional benefits compared to previous treatments for patients with schizophrenia and may enhance their quality of life. Further research is needed to assess the long-term impact of PP6M in the patient’s quality of life.

## Data Availability

The raw data supporting the conclusions of this article will be made available by the authors, without undue reservation.
